# Trends in type 2 diabetes-related deaths and disability-adjusted life years among smoking middle-aged and elderly adults in China, 1990–2021

**DOI:** 10.18332/tid/212546

**Published:** 2025-12-04

**Authors:** Yujun He, Chyuanan Lai, Bowen Xing, Hui Xu, Jiujie He, Wei Mai, Simin Qin, Jiajia Wang, Yuping Ye

**Affiliations:** 1 Department of Traditional Chinese Medicine, Taizhou Hospital of Zhejiang Province Affiliated to Wenzhou Medical University, Taizhou City, China; 2 Department of Traditional Chinese Medicine, The First People's Hospital of Chenzhou City, Chenzhou City, China; 3 Faculty of Acupuncture, Moxibustion and Tuina, Guangxi University of Chinese Medicine, Nanning City, China; 4 Department of Traditional Chinese Medicine, Guangxi Medical University Cancer Hospital, Nanning City, China; 5 Rehabilitation Medicine Department, The Second Affiliated Hospital of Hainan Medical University, Haikou City, China

**Keywords:** smoking, type 2 diabetes mellitus, deaths and disability-adjusted life years, China, ARIMA model prediction

## Abstract

**INTRODUCTION:**

Type 2 diabetes mellitus (T2DM) poses a global health crisis. Smoking, a key risk factor for T2DM, significantly impacts middle-aged and elderly populations. In China, with the world's largest elderly population and a high prevalence of smoking, the burden of smoking-related T2DM remains underrecognized.

**METHODS:**

Using GBD 2021 data, we applied joinpoint regression, age-period-cohort analysis, and the ARIMA model for prediction. This is a secondary dataset analysis. The study population included Chinese adults aged ≥55 years.

**RESULTS:**

From 1990 to 2021, deaths and DALYs attributable to smoking-associated T2DM showed a substantial increase, with males experiencing a significant rise in both number of death and DALYs rates, while females exhibited a decrease in death rate though total number of deaths rose. Joinpoint analysis revealed fluctuating trends in mortality and DALYs. The age-period-cohort analysis highlighted the age group of 70–75 years as a high-risk period. Predictive analysis suggested a slight upward trend in mortality for the overall population and males, while the DALYs rate was expected to remain stable but increase among males and decrease among females.

**CONCLUSIONS:**

From 1990 to 2021, the impact of smoking on type 2 diabetes mellitus (T2DM) among middle-aged and elderly adults in China continued to rise, with notable gender differences. Strengthening tobacco control and diabetes management, particularly for males and high-risk age groups, is crucial for reducing this burden.

## INTRODUCTION

Diabetes mellitus (DM) has become a global health crisis, affecting 537 million adults in 2021 and projected to reach 783 million by 2045^1^. Type 2 diabetes (T2DM), comprising 90–95% of diabetes, predominantly impacts middle-aged and elderly populations^2^. This chronic disorder reduces quality of life and imposes significant economic burdens, with rising mortality and disability-adjusted life years (DALYs) driven by complications like cardiovascular disease and renal failure^3^.

Smoking, a key risk factor for T2DM, influences pathogenesis through insulin signaling disruption, insulin resistance, and lipid metabolism alterations^4^. Epidemiological evidence consistently links smoking to higher T2DM incidence^5^. Middle-aged and elderly adults – with high T2DM prevalence and long-term smoking rates – face exacerbated disease severity and complication risks^6^. Despite this, global mortality/DALYs data for smoking-associated T2DM in this group remain scarce. The Global Burden of Disease (GBD) study offers a systematic framework to quantify this burden across regions.

In China– home to the world’s largest elderly population – T2DM prevalence is rising^7^, with smoking (27.7% male rate) as an underrecognized risk factor^8^. While prior GBD 2021 studies analyzed China’s T2DM burden^9,10^, smoking’s specific role remains unclear. Using GBD 2021 data and joinpoint regression/age-period-cohort analysis, this study examines smoking’s influence on T2DM burden in Chinese middle-aged and elderly adults (1990–2021), shedding light on 15-year trend dynamics. Understanding this burden is critical for prioritizing tobacco control, evaluating interventions (e.g. taxation, smoke-free policies), and targeting high-risk areas. This study addresses a major literature gap by analyzing smoking’s impact on deaths and DALYs of T2DM in Chinese aging populations.

## METHODS

### Research population and data compilation

The GBD 2021 study, with updated epidemiological data and enhanced standardization, quantified health loss from 371 diseases across 204 regions, using the metric such as death and DALYs^6^. Its dataset offers a thorough assessment of disease burdens. The GBD 2021 database classifies disease according to the International Classification of Diseases, 10th Revision (ICD-10). In the ICD-10, T2DM is categorized under the codes E11^11^. In the GBD study, the disease burden of smoking-associated T2DM is measured by deaths, DALYs, years of life lost (YLLs), and years lived with disability (YLDs). As DALYs integrate both YLLs and YLDs to simultaneously reflect the impacts of life loss and disability, we selected deaths and DALYs as the primary endpoints for analysis – all raw values of deaths and DALYs (and their rates) from GBD 2021 are reported with 95% UIs. The study population was defined as Chinese adults aged ≥55 years, and the time frame of the study was 1990–2021. This is a secondary dataset analysis.

Regarding exposure to risk factors, the GBD database systematically collates global epidemiological data and employs a comparative risk assessment framework to calculate risk factor exposure. This approach integrates diverse data sources, including population surveys, cohort studies, and environmental monitoring. It quantifies risk levels using the summary exposure value (SEV) and defines safety thresholds based on the theoretical minimum risk exposure level (TMREL). During the calculation process, a Bayesian meta-regression model is applied for data standardization, and stratified analyses are conducted by age, sex, and region. These steps ultimately generate comparable exposure assessments^12^.

### Data analysis

*Overview*

We began our data analysis by evaluating the dataset configuration to gauge the number and rate of deaths and DALYs resulting from smoking-associated T2DM in middle-aged and elderly Chinese adults. Subsequently, we examined the shifts in these measurements from 1990 to 2021. This assessment encompassed the deaths and DALYs cases and rates per 100000^13^. To determine the estimated annual percentage change (EAPC), we utilized the formula: EAPC = 100×(exp(β) - 1). The 95% confidence interval (CI) for EAPC was derived from a linear model. An increasing trend in rate metrics was suggested when both the EAPC value and the lower bound of the 95% CI surpassed zero. Conversely, a decreasing trend was observed when both the EAPC value and the upper bound of the 95% CI were beneath zero. A consistent trend was indicated if the 95% CI of EAPC encompassed zero^14,15^. To establish the relative changes (RCs), we applied the formula: RC (%) = [(value in 2021 - value in 1990)/value in 1990]×100%, which was also based on cases per 100000 individuals and rate^13^.

*Joinpoint regression analysis*

The joinpoint regression model consists of several linear statistical models used to evaluate the temporal variations in disease burdens of rates linked to smoking-associated T2DM in middle-aged and elderly Chinese adults. This model adopts a statistical approach that measures changes in disease incidence through the method of least squares, avoiding the subjectivity present in traditional trend evaluations that depend on linear trends^16^. The inflection point of the trend variation is determined by calculating the squared sum of the residual error between the estimated and actual values. The model was established using Joinpoint software (version 5.1.0.0; National Cancer Institute, Rockville, MD, USA), which analyzes temporal data patterns and then applies a basic model by connecting multiple line segments on a logarithmic scale. The annual percentage change (APC) was calculated to assess trends. The determination of the optimal number of joinpoints relied on a synergy of statistical criteria and model fit evaluation. The model selection procedure initiated with the minimum number of joinpoints (i.e. zero) and sequentially increased this number; the increment was halted when the introduction of additional joinpoints no longer yielded a significant improvement in model fit. Specifically, the built-in algorithm of Joinpoint software was employed, wherein a permutation test is integrated to assess the significance of each potential joinpoint. The software calculates the difference in the sum of squared residuals between the model with and without the additional joinpoint^17^.

*Age-period-cohort analysis*

In epidemiological research, age-period-cohort models are frequently employed to differentiate the effects of age, time period, and birth cohort on disease burden. These models, constructed on Poisson distributions, face an inherent identification problem – the parameters of age, period, and cohort cannot be uniquely estimated without additional constraints, as their effects are linearly dependent. To address this issue and ensure interpretable results, we applied the sum-to-zero constraint (a standard constraint in APC modeling) in our analysis: specifically, we set the sum of parameter estimates for each dimension (age groups, time periods, and birth cohorts) to zero. This constraint is consistent with the default setting of the age-period-cohort Web Tool (https://analysistools.cancer.gov/apc/) used for model fitting and aligns with conventional practices in disease burden research^18^. We analyzed the data from 1990 to 2021 in five-year cycles, calculating total deaths and DALYs for each age group.

*Predictive analysis*

To project future patterns in the deaths and DALYs burden from smoking-associated T2DM, we utilized the autoregressive integrated moving average (ARIMA) model. This model utilizes the autocorrelation within time-series data to project future values grounded in past observations. ARIMA is a commonly used time-series forecasting method; its core principle is to capture the autocorrelation (i.e. the inherent connection between data points at different time points) within time-series data. By analyzing the changing rules and trends of historical data on smoking-associated T2DM-related deaths and DALYs in this study, it infers and predicts the possible changes in the indicator in subsequent time periods. The fundamental concept of ARIMA is that data series are time-dependent random variables, which is defined by its autocorrelation structure. To function effectively, the time series needs to be stationary and stochastic, with a mean of zero^19^.

Prior to model fitting, we first conducted stationarity tests for the time-series data (deaths and DALYs rates) using the Augmented Dickey-Fuller (ADF) test. For non-stationary series, we performed differencing operations (determining the order of integration d) until the series met the stationary criterion (ADF test p<0.05). Next, we identified the optimal autoregressive order (p) and moving average order (q) by analyzing the autocorrelation function (ACF) and partial autocorrelation function (PACF) plots, and selecting the model with the minimum Akaike information criterion (AIC) and Bayesian information criterion (BIC) to balance fit and parsimony.

After model fitting, we conducted two key validation steps: 1) Residual analysis – the Ljung-Box test was used to verify whether the model residuals conformed to white noise (i.e. no remaining autocorrelation, indicating the model fully extracted time-series information), a p>0.05 was considered evidence of white noise; and 2) Predictive accuracy assessment – we evaluated model performance using three metrics, root mean square error (RMSE), mean absolute error (MAE), and mean absolute percentage error (MAPE), with smaller values indicating higher prediction accuracy. Predictive analyses were conducted using R version 4.3.3.

## RESULTS

### Overview of the global burden

*Trend analysis for deaths and DALYs burden of smoking-associated T2DM*

After preliminary inspection, no missing data were found, and the analysis proceeded smoothly. In 1990, the total number of deaths from T2DM attributable to smoking among Chinese adults aged ≥55 years was 5884.677 (95% UI: 4765.341–7185.385), with a rate of 4.100 per 100000 population (95% UI: 3.320–5.007). Specifically, males accounted for 4652.031 deaths (95% UI: 3659.188–5882.535), corresponding to a rate of 6.586 (95% UI: 5.180–8.327), while females had a total of 1232.646 deaths (95% UI: 924.974–1607.592) with a rate of 1.691 (95% UI: 1.269–2.206). By 2021, the total number of deaths increased to 14576.156 (95% UI: 11067.517–19128.553), representing a 147.70% increase from 1990, whereas the rate decreased by 6.19% to 3.846 (95% UI: 2.921–5.048). Among males, the total number of deaths rose by 173.57% to 12726.540 (95% UI: 9466.222–17019.134), with the rate increasing by 5.15% to 6.924 (95% UI: 5.150–9.260). In females, the total number of deaths increased by 50.05% to 1849.616 (95% UI: 1259.307–2585.228), accompanied by a 43.97% decrease in the rate to 0.948 (95% UI: 0.645–1.325). EAPCs showed no significant trend in the overall rate from 1990 to 2021 (-0.11; 95% CI: -0.29–0.08), while males exhibited an increasing trend (0.38; 95% CI: 0.24–0.52) and females showed a decreasing trend (-2.22; 95% CI: -2.67 – -1.78) (Table 1).

**Table 1:** Deaths and DALYs burden of smoking-associated T2DM, by sex, in middle-aged and elderly Chinese adults, 1990–2021

*Measure*	*Sex*	*1990* *number* *(95% UI)*	*1990* *rate* *(95% UI)*	*2021* *number* *(95% UI)*	*2021* *rate* *(95% UI)*	*EAPC* *(95% CI)*	*RC of* *number* *%*	*RC of* *rate* *%*
**Deaths**	Both	5884.677 (4765.341–7185.385)	4.100 (3.320–5.007)	14576.156 (11067.517–19128.553)	3.846 (2.921–5.048)	-0.11 (-0.29–0.08)	147.70	-6.19
	M	4652.031 (3659.188–5882.535)	6.586 (5.180–8.327)	12726.540 (9466.222–17019.134)	6.924 (5.150–9.260)	0.38 (0.24–0.52)	173.57	5.15
	F	1232.646 (924.974–1607.592)	1.691 (1.269–2.206)	1849.616 (1259.307–2585.228)	0.948 (0.645–1.325)	-2.22 (-2.67 – -1.78)	50.05	-43.97
**DALYs**	Both	287758.166 (225957.141–367908.015)	200.503 (157.442–256.350)	796463.374 (606212.385–1040119.365)	210.174 (159.969–274.470)	0.06 (0.01–0.12)	176.78	4.82
	M	237779.236 (184541.826–304633.679)	336.605 (261.241–431.246)	708999.809 (535494.158–929806.237)	385.759 (291.356–505.897)	0.42 (0.37–0.47)	198.18	14.60
	F	49978.930 (37502.957–65313.589)	68.579 (51.460–89.621)	87463.565 (59924.257–120204.021)	44.816 (30.705–61.592)	-1.77 (-2.00 – -1.53)	75.00	-34.65

M: male. F: female. RC: relative change. UIs are for GBD outputs, and CIs are for model-based estimates. ASR: age-standardized rate. RC: relative change. EAPC: estimated annual percentage change. UI: uncertainty interval. CI: confidence interval.

In 1990, the total number of DALYs from T2DM attributable to smoking among Chinese adults aged ≥55 years was 287758.166 (95% UI: 225957.141–367908.015), with a rate of 200.503 per 100000 (95% CI: 157.442–256.350). Specifically, males contributed 237779.236 (95% UI: 184541.826–304633.679), corresponding to a rate of 336.605 (95% CI: 261.241–431.246), whereas females had 49978.930 (95% UI: 37502.957–65313.589) with a rate of 68.579 (95% UI: 51.460–89.621). By 2021, the total DALYs increased to 796463.374 (95% UI: 606212.385–1040119.365), representing a 176.78% increase from 1990, while the rate rose by 4.82% to 210.174 (95% UI: 159.969–274.470). Among males, total DALYs surged by 198.18% to 708999.809 (95% UI: 535494.158–929806.237), with the rate increasing by 14.60% to 385.759 (95% CI: 291.356–505.897). In females, total DALYs increased by 75.00% to 87463.565 (95% UI: 59924.257–120204.021), accompanied by a 34.65% decrease in the rate to 44.816 (95% UI: 30.705–61.592). From 1990 to 2021, EAPCs showed an upward trend in the overall rate (0.06; 95% CI: 0.01–0.12), while males exhibited a significant increase (0.42; 95% CI: 0.37–0.47) and females showed a decline (-1.77; 95% CI: -2.00 – -1.53) (Table 1).

*Trends by year, sex and age for deaths and DALYs burden of smoking-associated T2DM*

**Figure 1:**
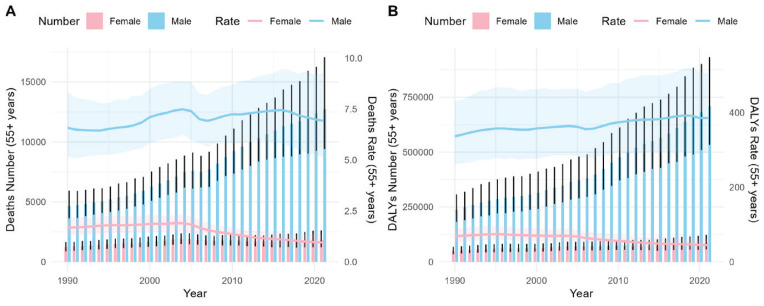
Deaths and DALYS, and age-standardized rates (per 100000 population) of females and males in China, 1990–2021. Bar represents number and the trend line represents rate. The shaded areas are 95% CI

As depicted in Figure 1, the number of deaths attributable to smoking among middle-aged and elderly Chinese adults (aged ≥55 years) with T2DM has shown a year-on-year increase. Although the change in the mortality rate per 100000 population is not very pronounced, it is significantly higher in males than in females. The trend of DALYs is similar to that of deaths.

**Figure 2:**
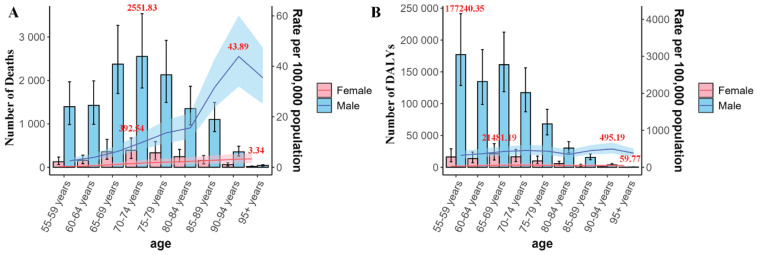
Trends in deaths and DALYS, and age-standardized rates (per 100000 population) of smoking-associated T2DM by gender and age group in China, 2021. The red font represents the maximum value. Bar represents number and the trend line represents rate. The shaded areas are 95% CI

Regarding the analysis by age groups, in terms of the number of deaths in 2021, there is an upward trend from the 55–59 years group to the 70–74 years group, with the latter group being the peak. After that, it decreases as the age group increases, and this characteristic is observed in both females and males. However, for the death rate in 2021, in males, there is a slow increase from the 55–59 years group to the 80–84 years group; from the 80–84 years group to the 90–94 years group, the growth suddenly accelerates, and then it starts to decline. In contrast, females have always been in a slow upward trend (Figure 2A). In terms of the absolute number of DALYs, both males and females show the same trend, that is, a general downward trend with the increasing age group, while the change in the rate is not obvious with the change of age groups (Figure 2B).

As can be seen from Supplementary file Table S1, in 1990, the age group with the highest number of deaths was the 65–69 years age group, while the lowest was the ≥95 years age group. The age group with the highest mortality rate was the 90–94 years age group, and the lowest was the 55–59 years age group. By 2021, changes occurred: the age group with the highest number of deaths became the 70–74 years age group. The 80–84 years age group had the largest increase in the EAPC, and the 60–64 years age group had the most significant decrease. The ≥95 years age group had the largest relative increase in the number of deaths, while the 60–64 years age group had the smallest change. The 85–89 years age group had the largest change in the death rate, and the 55–59 years age group had the most substantial relative decrease in the death rate.

Regarding DALYs, in 1990, the number of DALYs decreased with the increasing age group. The 70–74 years age group had the highest rate, and the ≥95 years age group had the lowest rate. By 2021, the change trends of the number and rate of DALYs with age groups were similar to those in 1990. The ≥95 years age group had the highest EAPC, and the 65–69 years age group had the lowest EAPC. The 60–64 years age group had the smallest relative change in the number of DALYs, while the ≥95 years age group had the largest relative change in the number of DALYs. The ≥95 years age group had the most significant decrease in the DALY rate, and the 75–79 years age group had the most obvious increase in the rate (Supplementary file Table S1).

### Joinpoint analysis

**Figure 3:**
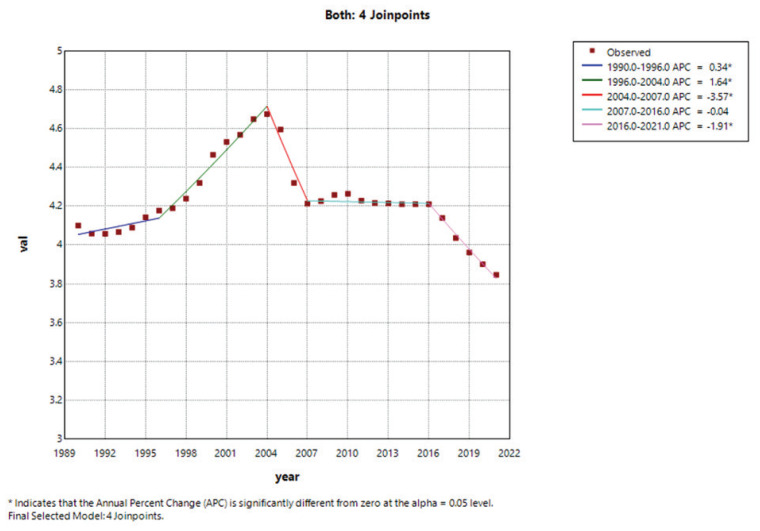
Joinpoint regression analysis of age-standardized DALYs rates for smoking-associated T2DM for both sexes in China, 1990–2021

The joinpoint analysis revealed that the overall (both sexes, Figure 3) AAPC from 1990 to 2021 was -0.19 (95% CI: -0.37 – -0.00), indicating that the mortality burden of smoking among middle-aged and elderly patients with T2DM aged ≥55 years did not show a statistically significant changing trend. This trend was divided into five distinct phases: 1990–1996 (APC=0.34; 95% CI: 0.04–0.64), 1996–2004 (APC=1.64; 95% CI: 1.42–1.87), 2004–2007 (APC= -3.57; 95% CI: -5.10 – -2.01), 2007–2016 (APC= -0.04; 95% CI: -0.22–0.15), and 2016–2021 (APC= -1.91; 95% CI: -2.41– -1.40). Except for the period of 2007–2016, all phases showed significant trends (p<0.05). The temporal pattern in males was generally consistent with the overall trend, while females exhibited a trend of first increasing and then decreasing (Supplementary file Tables S1 and S2, Figures S1A–S1C).

The results of DALYs showed a more complex trend. Overall, both sexes and males exhibited an upward trend, while females showed a downward trend (Supplementary file Table S2 and Figures S1D–S1F).

*Age-period-cohort analysis*

For age-related effects, Supplementary file Figure S2A shows that mortality rates were lower than expected before 65 years of age and after 80 years, while mortality between 65 and 80 years exceeded expectations, peaking particularly in the age group of 70–75 years. This underscores the necessity of focusing attention on these age segments with exceeded expectations. Supplementary file Figure S2B indicates that age itself acts as a promoting factor for mortality, as the curve shows a continuous upward trend with increasing age. When compared with Supplementary file Figure S2B, the cross-sectional observations in Supplementary file Figure S2C also confirm that mortality rises with age.

Regarding period-related effects, Supplementary file Figure S2D reflects the influence of external factors across different periods. The fluctuating curve indicates that the period is a significant confounding factor, as evident from the notable deviations in certain years (every 5 years starting from 2000), which may correspond to medical reforms or public health events. The overall downward-trending fit of the temporal trend in Supplementary file Figure S2E suggests improvements in population health outcomes. Supplementary file Figure S2F (Period RR) quantifies the relative risk (RR) across periods; curves below 1 after 2005 indicate that the risk in the corresponding periods is lower than the average level, whereas the opposite is true for the results before this time.

For cohort-related effects, Supplementary file Figure S2G shows outcome deviations among different birth cohorts, and the shape of the curve reflects cohort-specific influences. Larger deviations are seen in earlier cohorts (left side), while later cohorts (right side) tend to exhibit a more stable trend. Supplementary file Figure S2H (Cohort RR) quantifies the relative risk for each cohort; the consistently low RR for cohorts from 1940 onward indicates a lower long-term risk.

From the age-period-cohort analysis of DALYs, it can be seen that the results regarding period and cohort are similar to those of mortality (Supplementary file Figures S3D–S3H). For the age-related results, the age range of approximately 70–75 years remains a high-risk period (Supplementary file Figure S3A). However, both in the longitudinal (Supplementary file Figure S3B) and cross-sectional (Supplementary file Figure S3C) perspectives, it is shown that DALYs first increase with age, reach a peak between 70 and 75 years, and then decline rapidly.

*Predictive analysis*

We predicted the disease burden over the next 15 years in terms of the mortality rate and the DALYs rate among middle-aged and elderly patients with T2DM aged ≥55 years caused by smoking. Model validation confirmed the reliability of the ARIMA models for forecasting. For all outcomes (deaths and DALYs by sex), the Ljung-Box test yielded p>0.05 (range: 0.201–0.928; Supplementary file Table S3), indicating that residuals were white noise and no unextracted autocorrelation remained. Additionally, the models exhibited low values of AIC (range: -124.860–145.368), BIC (range: -123.46–151.104), RMSE (range: 0.016–4.695), MAE (range: 0.013–3.585), and MAPE (range: 0.925–4.816%; Supplementary file Table S3), further confirming good fit and predictive accuracy – particularly for female mortality, which showed the lowest MAPE (1.31%).

Over the next 15 years, in terms of mortality, both the overall population and males showed a slight upward trend, while the mortality rate among females was projected to decline significantly. Regarding the DALYs rate, the overall trend was expected to remain stable, yet it would increase among males and decrease among females (Supplementary file Figure S4).

## DISCUSSION

### Trends and gender differences in smoking-associated deaths and DALYs of T2DM

The present study comprehensively analyzed the trend of deaths and DALYs burden caused by smoking-associated T2DM among middle-aged and elderly Chinese adults from 1990 to 2021, revealing several noteworthy findings.

Firstly, the total number of deaths showed a remarkable increase over the three decades. This growing mortality burden might be attributed to the rising prevalence of smoking in the older population and the prolonged exposure time to smoking, which exacerbates the risk of T2DM and its complications^20^. Additionally, with the aging of the population, there is a larger base of middle-aged and elderly individuals susceptible to smoking-associated diseases, further contributing to the increased number of deaths^21^. However, it is interesting to note that the overall death rate exhibited a slight decrease despite the substantial rise in the total number of deaths. This could be explained by the advances in medical technology and healthcare services, which have improved the diagnosis and treatment of T2DM and its complications, thereby reducing the case-fatality rate^22^. Moreover, public health interventions aimed at smoking cessation and diabetes management may have played a role in curbing the death rate.

When examining the gender-specific trends, a stark contrast emerged. Males experienced a significant increase in both the number of deaths and the death rate, which is in line with the fact that male smoking rates have historically been much higher than those of females in China^8^. The deeply ingrained smoking culture among Chinese males has led to a larger proportion of male smokers and a longer duration of smoking, making them more vulnerable to smoking-associated T2DM and its fatal consequences. On the other hand, females showed a substantial increase in the total deaths but a marked decrease in the death rate. This seemingly contradictory pattern might be due to the rapid increase in the female population aged ≥55 years during the study period, leading to more smoking-associated T2DM cases and deaths in absolute numbers. However, the relatively lower smoking prevalence in females, coupled with potentially better healthcare-seeking behaviors and diabetes management practices, or biological factors compared to males, could have contributed to the decline in the death rate^23^.

Regarding the DALYs burden, a similar trend was observed. The total DALYs attributable to smoking among Chinese diabetic patients aged ≥55 years increased dramatically from 1990 to 2021, reflecting the growing disease burden imposed by smoking-associated T2DM. This increase in DALYs indicates not only the higher mortality but also the increased years lived with disability caused by smoking-related T2DM complications. The rising DALYs rate, especially among males, highlights the severe impact of smoking on the quality of life and productivity of middle-aged and elderly individuals. The gender disparity in DALYs trends further emphasizes the differential burden of smoking-associated T2DM between males and females.

The EAPCs analysis provided valuable insights into the long-term trends. The absence of a significant trend in the overall death rate but an upward trend in the overall DALYs rate suggests that while the risk of death from smoking-associated T2DM has not changed substantially, the overall disease burden in terms of DALYs has increased. This could be due to improved survival rates among patients with smoking-associated T2DM, leading to more years lived with disability. For males, the increasing EAPCs for both death rate and DALYs rate underscore the urgent need for targeted interventions to reduce smoking-related T2DM burden in this high-risk group. In contrast, the decreasing EAPCs for females indicate some positive progress in reducing the burden among females, although the absolute number of deaths and DALYs still remains a concern (gender differences: for the two indicators– deaths and DALYs – the p-values of both the interaction terms and the Z-tests were all <0.0001).

These findings have several important implications. Firstly, we emphasize the critical need for enhanced smoking cessation programs and diabetes prevention strategies tailored to middle-aged and elderly populations. Given the higher smoking prevalence and greater burden among males, special attention should be directed towards male smokers. Public health campaigns should focus on raising awareness of the link between smoking and T2DM, as well as the severe consequences of smoking-associated T2DM. Secondly, the results highlight the importance of improving healthcare services for smoking-associated T2DM patients. This includes early detection, timely treatment, and effective management of diabetes and its complications to reduce mortality and disability^24^. Thirdly, the gender-specific trends suggest that gender-sensitive approaches should be adopted in public health policies and interventions. For females, efforts should focus on preventing the uptake of smoking and promoting healthy lifestyles, while for males, more intensive smoking cessation support and regular health check-ups are warranted.

### Synthesis of gender and age trends

The escalating numbers of deaths and DALYs attributable to smoking-associated T2DM underscore the growing health burden of this disease. Although the overall mortality rate remained non-significant, males consistently exhibited a significantly higher mortality number and rate than females.

Age-stratified analysis revealed that the age group of 70–74 years witnessed the peak number of deaths in 2021, with an upward trend from 55 to 74 years, followed by a decline among older age groups – a pattern consistent across males and females. Male mortality rates increased gradually from the 55–59 years age group, accelerated after the age group of 80–84 years, and subsequently declined from 90–94 years. In contrast, female mortality rates showed a steady, albeit slow, increase across all age groups. These findings suggest that older adults, particularly men, face a disproportionately higher per-capita mortality risk, potentially attributable to the cumulative effects of lifelong smoking exposure^21^. Regarding DALYs, the absolute numbers decreased with advancing age in both genders, while the rates remained relatively stable, indicating that although the overall disease burden lessens with age, the incidence of disability remains constant or fluctuates depending on factors such as access to geriatric healthcare and quality of life.

Comparing data from 1990 and 2021, the age group with the highest number of deaths shifted from 65–69 to 70–74 years. The age group of 80–84 years exhibited the most substantial increase in the EAPC, whereas the 60–64 years age group showed the most significant decline. Notably, the age group ≥95 years experienced the highest relative increase in deaths, while the age group of 55–59 years demonstrated the most pronounced relative decrease in mortality rates. For DALYs, the trends in both the number and rate changes remained similar between the two years, with the age group of ≥95 years having the highest EAPC and the age group of 75–79 years showing the most notable rate increase.

These findings have profound implications for public health. First, tailored smoking cessation and diabetes prevention strategies targeting middle-aged and older men are imperative, accompanied by enhanced public awareness campaigns highlighting the link between smoking and T2DM^25^. Second, given the elevated burden in the age group of 70–74 years, resources should prioritize early detection, timely treatment, and comprehensive disease management for this high-risk population^26^. Third, preventive measures must be intensified among younger age groups to curb disease progression effectively. The marked variations in EAPC across age groups further emphasize the need for dynamic public health strategies that adapt to the evolving burden, particularly among the elderly.

Future research should elucidate the biological, medical, and socioeconomic determinants underlying the observed gender disparities and temporal trends. Evaluating the effectiveness of existing smoking cessation programs and diabetes management interventions, especially in middle-aged and older adults, and conducting longitudinal studies and randomized controlled trials will provide crucial evidence for formulating more impactful public health strategies.

### Joinpoint analysis of smoking-associated mortality and DALYs of T2DM

Regarding mortality, the overall AAPC of -0.19 (95% CI: -0.37 – -0.00) indicates no significant overall trend. However, the five distinct phases show fluctuations, including increases and decreases, with only the 2007–2016 period showing no significant trend. These fluctuations might result from changing smoking patterns, public health policies, and healthcare advances over time. For instance, the increase in the 1990s could be due to the lagged effect of smoking on T2DM development, as smoking rates in China were relatively high during that period^27^. The decrease after 2004 might reflect the impact of initial tobacco control policies and improved diabetes management^28^. Since the World Health Organization’s Framework Convention on Tobacco Control (FCTC) came into effect in China in January 2006, significant progress has been made in tobacco control, including monitoring tobacco use, implementing prevention policies, protecting people from tobacco smoke, promoting smoking cessation, warning about tobacco hazards, conducting anti-tobacco media campaigns, using health warning labels, banning tobacco advertising and sponsorship, and increasing tobacco taxes^29^. The lack of a significant trend from 2007 to 2016 likely indicates a period of relative stability in smoking prevalence. After this period, the positive effects of tobacco control became more evident, leading to a downward trend in mortality.

For DALYs, the overall increasing trend for both sexes and males contrasts with the decreasing trend for females. This divergence may result from the combined effects of smoking cessation, better healthcare access, and improved disease management in females, whereas males remain heavily affected by higher smoking rates and related complications.

These results highlight the dynamic nature of the smoking-associated T2DM burden and emphasize the importance of adaptable public health strategies. Effective tobacco control and diabetes prevention policies must consider these temporal variations and gender differences. Future research should investigate the specific factors driving these trends in different periods. This can inform more targeted and timely interventions to reduce the burden of smoking-associated T2DM in this population.

### Age-period-cohort analysis of smoking-associated mortality and DALYs of T2DM

For age-related effects, the elevated mortality and DALYs observed in the age group of 70–75 years are particularly relevant in the context of T2DM and smoking in China. Middle-aged and elderly Chinese adults in this age bracket often face a double burden of smoking-associated health risks and T2DM complications. The higher than expected mortality between 65 and 80 years highlights the urgent need for targeted smoking cessation programs and enhanced T2DM management strategies tailored to this vulnerable age segment in China. Given the country’s large aging population, effectively addressing these issues can significantly reduce the disease burden.

The period-related effects reveal the dynamic impact of external factors on T2DM-attributable deaths and DALYs among Chinese middle-aged and elderly smokers. The fluctuations in the period curve, especially the deviations in certain years, likely mirror the influence of China’s evolving public health policies, economic development, and changes in smoking prevalence. This might be due to factors such as the introduction of new tobacco products, changes in smoking patterns, or challenges in the enforcement of tobacco-control policies in recent years^30^. For example, the implementation of anti-smoking regulations and improved diabetes care initiatives might have contributed to the observed trends^29,31^. The overall downward trend in the temporal trend curve suggests that China’s continuous efforts in public health, such as promoting healthy lifestyles and improving medical services, have led to positive improvements in population health outcomes related to T2DM and smoking. However, the relative risk quantification across periods indicates that there is still room for improvement, particularly in earlier years when risk levels were higher.

Regarding cohort-related effects, the differences among birth cohorts in China reflect the changing social, economic, and health environments over time. Earlier cohorts may have been exposed to higher smoking rates due to less awareness of its harms and fewer anti-smoking measures, while later cohorts benefit from increased health education and stricter tobacco control policies^29^. The consistently low relative risk for cohorts from 1940 onward implies that younger generations of middle-aged and elderly Chinese adults are at a lower long-term risk of T2DM-attributable deaths and DALYs associated with smoking, which is a positive outcome of China’s continuous health promotion efforts^31^. However, the still existing risk underscores the need for sustained interventions.

The similarities in the period and cohort results between mortality and DALYs analyses further emphasize the comprehensive impact of smoking on T2DM-related health outcomes in Chinese middle-aged and elderly adults. The unique pattern of DALYs, with a rapid decline after the peak in the age group of 70–75 years, may be related to the complex relationship between smoking, T2DM, and other comorbidities. It could also be influenced by differences in the quality of life and healthcare access among different age groups in China.

### Predictive analysis of smoking-associated mortality and DALYs of T2DM

The divergent trends in mortality between genders are particularly noteworthy. The projected slight upward trend in overall and male mortality, coupled with the significant decline in female mortality, may be attributed to several factors specific to the Chinese context. In China, although smoking prevalence has been gradually decreasing over the years, males still constitute the majority of smokers, and smoking remains a deeply ingrained habit in male-dominated social and cultural environments^8^. For middle-aged and elderly male T2DM patients, the combined effects of long-term smoking exposure and the progression of T2DM may lead to an increased risk of severe complications, such as cardiovascular diseases and respiratory failures, contributing to the upward mortality trend^32^. On the other hand, the decline in female mortality could be a result of more effective smoking cessation campaigns targeted at women in recent years, as well as improved access to diabetes management and healthcare services for females. Additionally, the cultural and social changes that encourage healthier lifestyles among women might also play a role in reducing the disease burden associated with smoking and T2DM.

Regarding the DALYs rate, the stable overall trend masks the contrasting changes between genders. The projected increase in the DALYs rate among males indicates that, despite potential improvements in some aspects of healthcare, the long-term impact of smoking on male T2DM patients will continue to pose a significant challenge to their quality of life and functional ability. This could be due to the persistence of smoking-associated comorbidities and the relatively slower progress in implementing comprehensive male-oriented health promotion programs. Conversely, the decrease in the DALYs rate among females aligns with the decline in mortality and further suggests that the current public health efforts targeting women, such as promoting smoking cessation and enhancing diabetes care, are having a positive impact on reducing both the burden of premature death and years lived with disability.

These findings have important implications for public health policy-making in China. Given the projected trends, it is imperative to strengthen gender-specific interventions. For males, more aggressive smoking cessation strategies, integrated with enhanced T2DM management programs, should be implemented to curb the upward trend in mortality and DALYs rate. This could include targeted anti-smoking education in male-dominated workplaces and communities^29^, as well as the development of male-tailored diabetes treatment and prevention plans. For females, while the current positive trends are encouraging, continuous efforts are needed to sustain and further enhance the effectiveness of existing health initiatives.

### Public health policy recommendations

In light of the research findings on the burden of smoking-associated type 2 diabetes mellitus (T2DM) among middle-aged and elderly Chinese adults, several public health policy recommendations can be put forward. First, tobacco control policies should be further strengthened, with a focus on reducing smoking prevalence, especially among male smokers. This can be achieved through increasing tobacco taxes, enhancing anti-smoking media campaigns, and strengthening the enforcement of tobacco advertising bans^33^. Second, comprehensive diabetes prevention and management programs should be improved, including promoting healthy lifestyles, enhancing early detection and diagnosis of T2DM, and providing timely and effective treatment and disease management for patients. Third, gender-sensitive and age-tailored strategies are essential. For males, more intensive smoking cessation support and regular health check-ups are needed, while for females, efforts should focus on preventing the uptake of smoking and promoting healthy lifestyles^34^. Special attention should be paid to the high-risk age group of 70–74 years, with resources prioritized for early detection, timely treatment, and comprehensive disease management. Finally, continuous evaluation of the effectiveness of existing smoking cessation programs and diabetes management interventions, as well as conducting more longitudinal studies and randomized controlled trials, will help to formulate more targeted and effective public health policies to reduce the burden of smoking-associated T2DM in this population.

### Limitations

Despite the valuable insights provided by this study, several limitations should be acknowledged. Firstly, the analysis primarily relied on historical data, which may not fully account for future changes in China’s social, economic, and healthcare landscapes. For instance, emerging smoking patterns, such as the increasing popularity of e-cigarettes, or breakthroughs in T2DM treatment could significantly alter the projected trends and disease burden^35^. Secondly, although multiple statistical methods were employed, the models used, such as the ARIMA model in predictive analysis, made certain assumptions about the data’s stability and trends. These models might not comprehensively capture the complex interactions between smoking, T2DM, and other confounding factors, such as genetic predispositions and environmental exposures. Additionally, residual confounding may exist due to unmeasured individual factors (e.g. smoking intensity, diet) in population-level GBD data. As an ecologic analysis, it risks ecologic fallacy when inferring individual-level associations. ARIMA models, though validated, cannot account for external covariates or unforeseen events, limiting long-term projection accuracy. We lacked subgroup analyses by region and data on e-cigarette use, which may mask regional disparities and underestimate the total smoking-related burden amid growing e-smoking. Also, reliance on historical GBD data may not reflect future societal or healthcare changes. Finally, the study focused on middle-aged and elderly Chinese adults as a whole, without delving deeply into subgroup differences within this population, such as variations in disease burden across different regions or socioeconomic statuses. This lack of detailed subgroup analysis may obscure more nuanced trends and needs. Recognizing these limitations can guide future research efforts to improve the understanding of the relationship between smoking and T2DM in this population.

## CONCLUSIONS

This study reveals that from 1990 to 2021, the number of smoking-associated T2DM deaths and DALYs among middle-aged and elderly Chinese adults increased significantly, with males bearing a heavier burden and a peak in the 70–74 age group. Predictions show a slight upward trend in overall and male mortality and DALYs rates in the next 15 years, while females may see a decline. Strengthening tobacco control, improving T2DM management, and implementing gender- and age-specific interventions are crucial to reducing this disease burden.

## Supplementary Material



## Data Availability

The data supporting this research are available from the following source: https://vizhub.healthdata.org/gbd-results/.
