# Supplementary material for: Trends in type 2 diabetes-related deaths and disability-adjusted life years among smoking middle-aged and elderly adults in China, 1990–2021
Source: Tob Induc Dis. 2025 Dec 4;23:10.18332/tid/212546. doi: 10.18332/tid/212546 (PMC12676998; doi:10.18332/tid/212546)
Supplement: Supplementary file 1 [file TID-23-190-s1.pdf]

Table S1 Deaths and DALYs Burden of Smoking-associated T2DM in Middle-Aged and Elderly Chinese Adults by Different Age Groups, 1990–2021.

Measure	Age Group	1990 number of years (95% UI)	1990 rate (95% UI)	2021 number of years (95% UI)	2021 rate (95% UI)	EAPC (95% CI)	RC of numbers (%)	RC of rate (%)
Deaths	55-59 years	920.409(708.501-1148.574)	2.122(1.634-2.648)	1524.213(1102.187-2112.762)	1.386(1.003-1.922)	-1.35(-1.47,-1.22)	65.6	-34.67
	60-64 years	1055.968(833.468-1348.335)	2.988(2.359-3.816)	1583.263(1138.388-2180.265)	2.169(1.559-2.986)	-0.88(-0.99,-0.76)	49.93	-27.43
	65-69 years	1274.173(995.572-1573.600)	4.670(3.649-5.768)	2731.836(1990.418-3695.330)	3.562(2.595-4.818)	-0.79(-0.96,-0.61)	114.4	-23.74
	70-74 years	1215.898(961.795-1494.252)	6.461(5.111-7.941)	2944.367(2158.969-4027.620)	5.525(4.051-7.557)	-0.38(-0.54,-0.21)	142.16	-14.5
	75-79 years	804.356(612.783-1026.525)	7.068(5.384-9.020)	2461.521(1772.711-3287.665)	7.432(5.353-9.927)	0.51(0.23,0.78)	206.02	5.16

	80-84 years	375.909(286.890-485.581)	7.096(5.416-9.167)	1594.247(1164.806-2141.590)	8.055(5.885-10.821)	0.62(0.34, 0.91)	324.1	13.51
	85-89 years	189.117(144.669-240.475)	11.211(8.576-14.256)	1257.446(941.777-1650.759)	13.200(9.887-17.329)	0.59(0.19, 0.98)	564.9	17.74
	90-94 years	44.779(33.730-56.563)	14.595(10.993-18.435)	419.087(304.122-566.830)	14.294(10.373-19.333)	-0.03(-0.25, 0.20)	835.89	-2.06
	95+ years	4.068(2.869-5.459)	10.047(7.086-13.481)	60.175(41.762-80.687)	9.416(6.534-12.625)	-0.34(-0.83, 0.15)	1379.12	-6.29
DALYs	55-59 years	72590.753(55185.315-94929.540)	167.379(127.246-218.887)	193457.321(140182.458-265535.739)	175.962(127.505-241.523)	0.11(0.05, 0.16)	166.5	5.13
	60-64 years	69443.139(53216.364-91297.926)	196.514(150.595-258.360)	148389.838(109413.781-202097.356)	203.259(149.871-276.826)	0.00(-0.05, 0.06)	113.69	3.43
	65-69 years	64875.176(50361.903-84689.853)	237.796(184.599-310.426)	182682.189(134308.118-241066.055)	238.167(175.100-314.283)	-0.11(-0.20, -0.03)	181.59	0.16
	70-74	46225.583(35511.456-59242.829)	245.650(188.714-314.826)	133646.594(99194.337-179838.563)	250.761(186.118-337.431)	0.01(-	189.12	2.08

years					0.07, 0.09)		
75-79 years	23006.568(17493.355-29232.494)	202.154(153.711-256.860)	78269.673(58476.647-104456.492)	236.329(176.566-315.398)	0.60(0.41, 0.78)	240.21	16.91
80-84 years	8230.466(6059.567-10668.319)	155.376(114.393-201.398)	35860.799(26562.281-46805.511)	181.190(134.208-236.489)	0.43(0.22, 0.64)	335.71	16.61
85-89 years	2818.464(2119.924-3625.584)	167.084(125.673-214.932)	18502.645(13839.985-24091.574)	194.238(145.290-252.910)	0.36(0.08, 0.64)	556.48	16.25
90-94 years	521.324(393.031-669.800)	169.911(128.097-218.302)	4969.943(3626.133-6582.368)	169.508(123.675-224.502)	-0.13(-0.33, 0.06)	853.33	-0.24
95+ years	46.692(32.981-62.588)	115.313(81.452-154.569)	684.372(480.236-922.320)	107.084(75.143-144.316)	-0.54(-1.00, -0.08)	1365.7	-7.14

Abbreviations: ASR: age-standardized rate; **RC:** relative change; **EAPC:** estimated annual percentage change; **SDI:** sociodemographic index; **UI:** uncertainty interval; **CI:** confidence interval.

Note: UIs are for GBD outputs, and CIs are for model-based estimates.

Table S2 Joinpoint Regression Analysis of Smoking - Induced T2DM Deaths and DALYs Burden Rates (per 100,000 Person - Years) Among Middle - aged and Elderly Chinese Adults by Sex, 1990 - 2021.

Gender	Deaths			DALYs		
	period	APC (95% CI)	AAPC (95% CI)	period	APC (95% CI)	AAPC (95% CI)
Both	1990-1996	0.34 (0.04 - 0.64)	-0.19 (-0.37 - 0.00)	1990-1995	1.45 (1.07 - 1.83)	0.18 (-0.10 - 0.45)
	1996-2004	1.64 (1.42 - 1.87)		1995-1998	-0.70 (-2.37 - 1.00)	
	2004-2007	-3.57 (-5.10 - -2.01)		1998-2004	0.32 (-0.04 - 0.69)	
	2007-2016	-0.04 (-0.22 - 0.15)		2004-2007	-1.30 (-2.90 - 0.33)	
	2016-2021	-1.91 (-2.41 - -1.40)		2007-2010	1.03 (-0.65 - 2.74)	
				2010-2021	-0.06 (-0.19 - 0.06)	
Male	1990-1995	-0.27 (-0.74 - 0.19)	0.18 (-0.09 - 0.45)	1990-1995	1.77 (1.50 - 2.03)	0.44 (0.21 - 0.67)
	1995-2004	1.78 (1.57 - 1.98)		1995-2000	-1.40 (-1.74 - -1.07)	
	2004-2007	-2.97 (-4.65 - -1.25)		2000-2004	-0.09 (-0.62 - 0.45)	
	2007-2010	1.63 (-0.17 - 3.46)		2004-2008	-3.87 (-4.39 - -3.35)	

	2010-2016	0.48 (0.01 - 0.95)		2008-2014	-2.87 (-3.12 - - 2.61)	
	2016-2021	-1.52 (-2.10 - - 0.94)		2014-2021	-1.48 (-1.67 - - 1.30)	
Female	1990-2004	0.89 (0.79 - 0.99)	-1.87 (-2.08 - - 1.66)	1990-1993	1.61 (0.83 - 2.39)	-1.36 (-1.48 - -1.23)
	2004-2007	-6.59 (-8.40 - - 4.75)		1993-2004	0.24 (0.12 - 0.37)	
	2007-2013	-4.51 (-4.93 - - 4.09)		2004-2007	-0.52 (-2.01 - 0.98)	
	2013-2021	-2.80 (-3.09 - - 2.51)		2007-2010	1.64 (0.08 - 3.22)	
				2010-2018	0.54 (0.32 - 0.76)	
				2018-2021	-0.51 (-1.36 - 0.36)	

Abbreviations: AAPC: average annual percent change; **APC:** annual percent change; **CI:** confidence interval.

Table S3 Validation of ARIMA's suitability for long-term predictions

Measure	Sex	AIC	BIC	LB p value	RMSE	MAE	MAPE
Deaths	Both	-85.72935123	-78.40067171	0.928277648	0.24229845	0.189399851	4.816258085
	Male	-58.22512313	-55.35714872	0.607759098	0.273791009	0.214761679	3.044257318
	Female	-124.8608889	-123.4596915	0.462173476	0.015697462	0.013171983	1.309274523
DALYs	Both	119.1248816	126.4535612	0.297340289	3.003576429	2.315398397	1.078524072
	Male	145.3680839	151.1040328	0.833664539	4.69541503	3.584567711	0.925351496
	Female	60.80003154	62.20122892	0.201358752	2.2743578	2.020928801	4.358382157

AIC: Akaike Information Criterion, **BIC:** Bayesian Information Criterion, **LB p value:** Ljung-Box Test p-value, **RMSE:** Root Mean Square Error, **MAE:** Mean Absolute Error, **MAPE:** Mean Absolute Percentage Error.

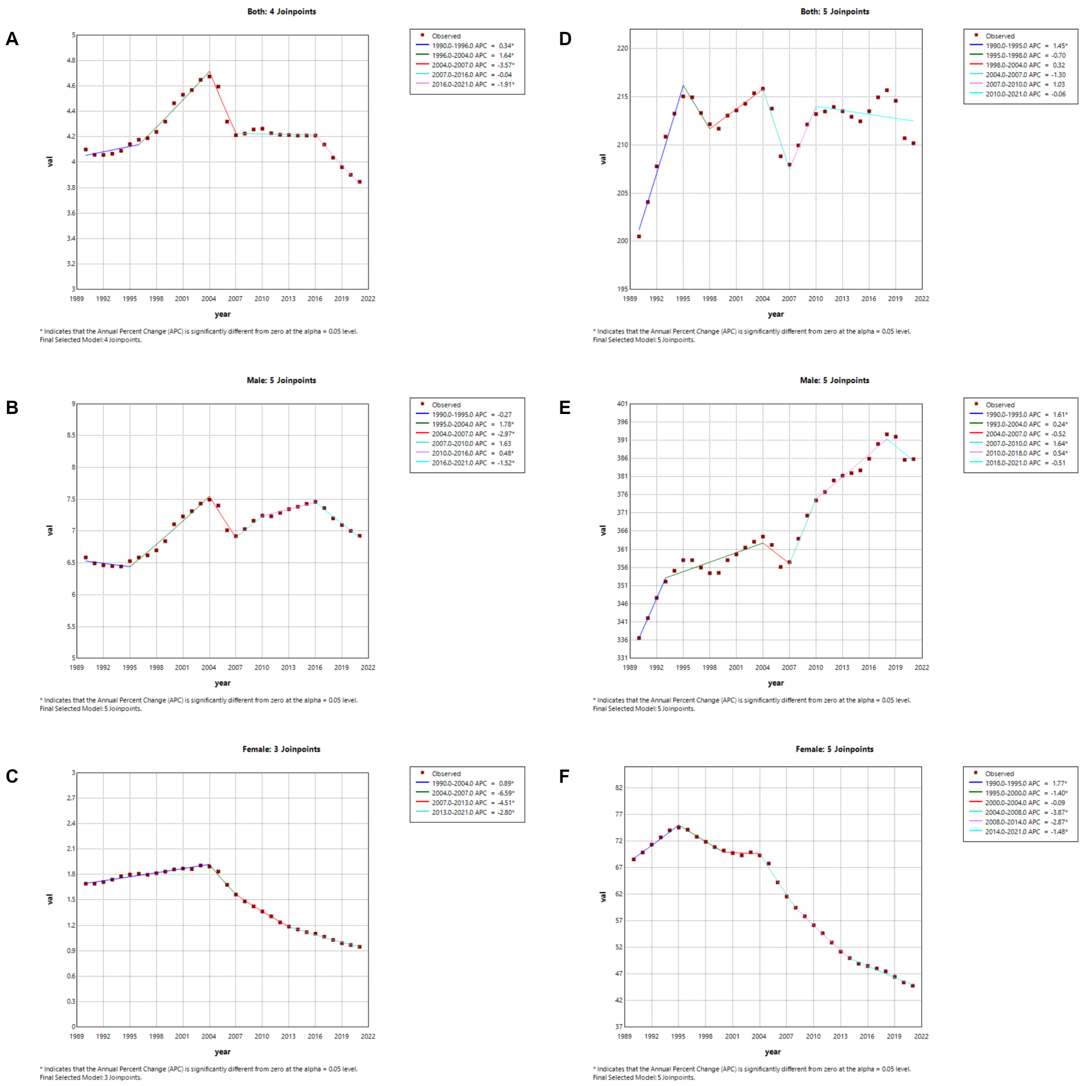

Figure S1 Joinpoint regression analysis of sex-specific age-standardized DALYs rates for smoking-associated T2DM in China: a study spanning 1990 to 2021 (a) both sexes, (b) males, (c) females.

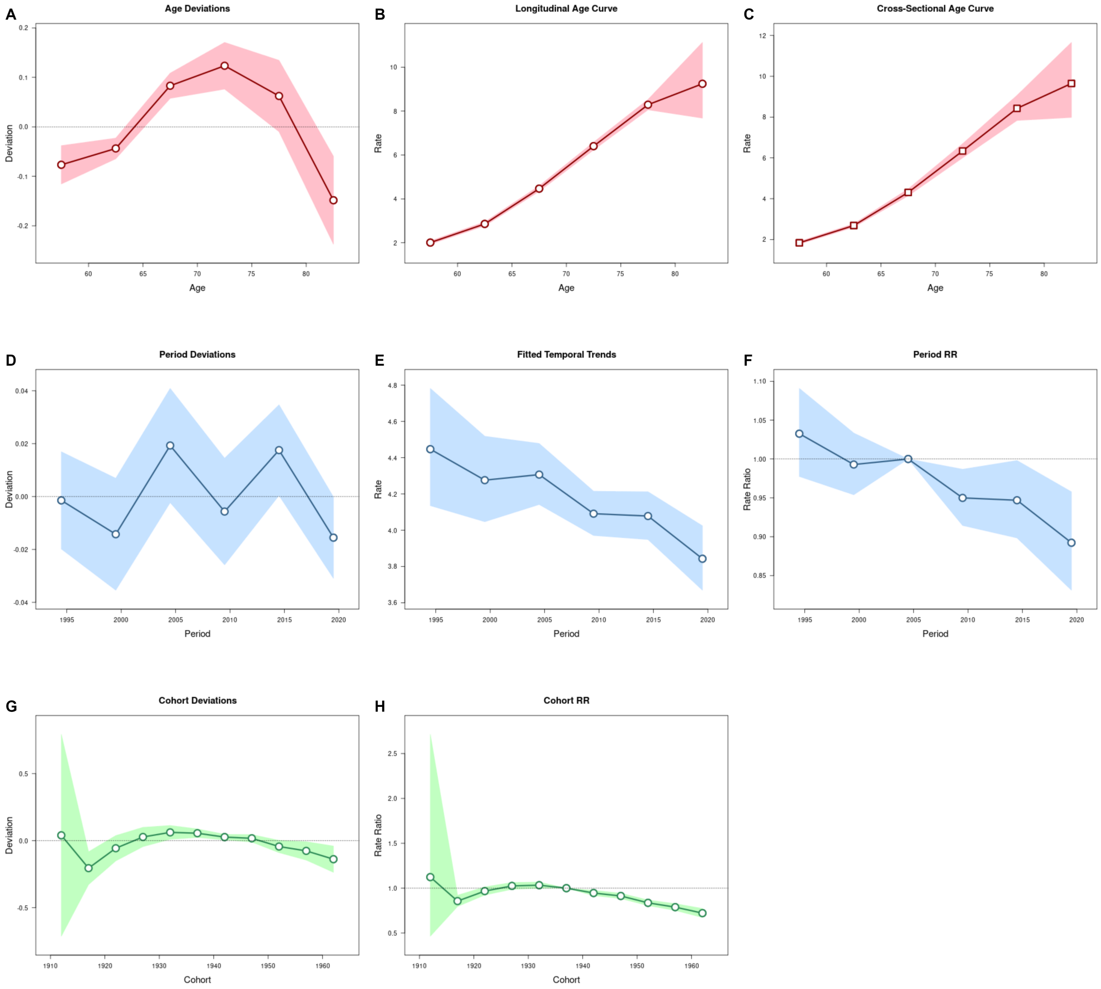

Figure S2 Smoking-associated T2DM - Related Deaths Burden Among Middle - aged and Elderly Chinese Adults: Results of Age - Period - Cohort Analysis Displaying Trends and Deviations in Rates across Age, Period, and Cohort Dimensions. (A) Age Deviations; (B) Longitudinal Age Curve; (C) Cross - Sectional Age Curve; (D) Period Deviations; (E) Fitted Temporal Trends; (F) Period RR; (G) Cohort Deviations; (H) Cohort RR.

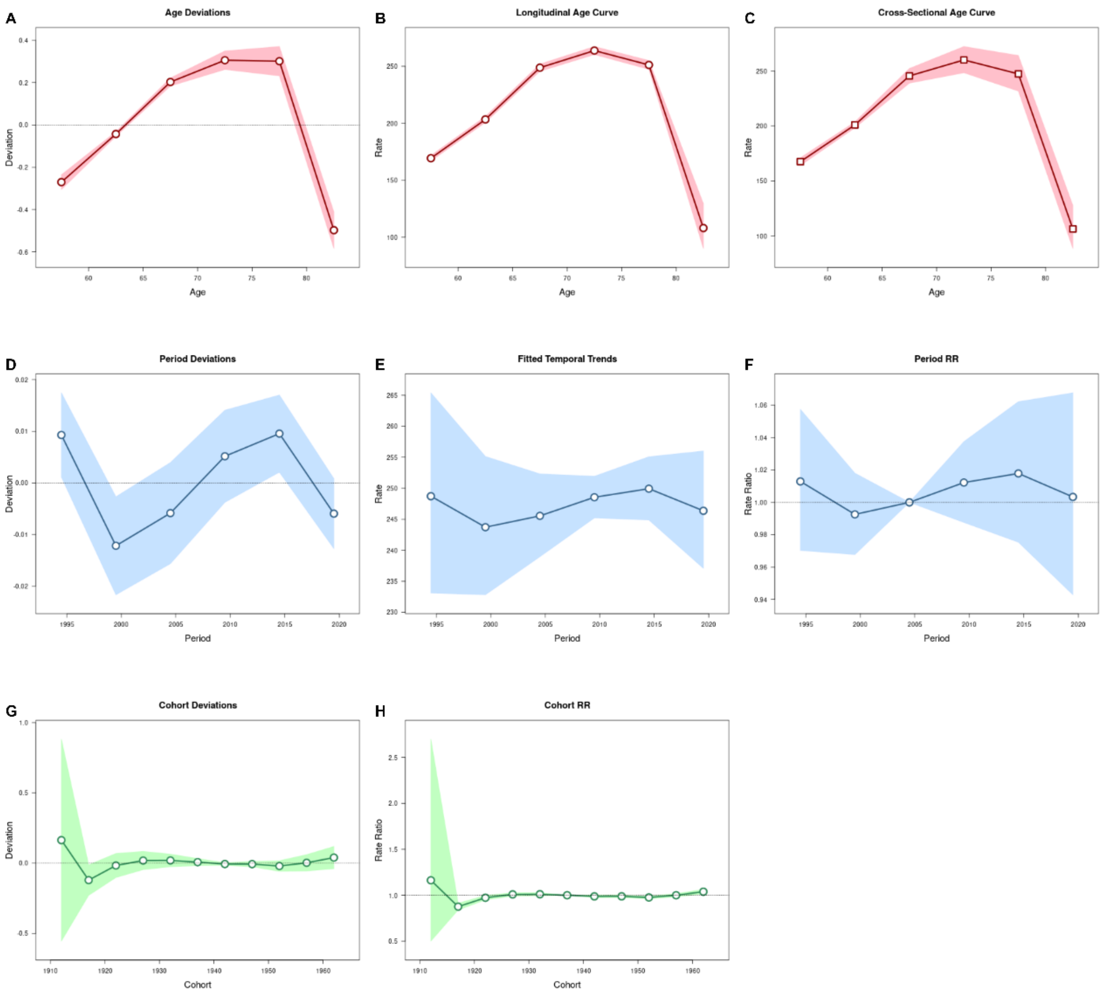

Figure S3 Smoking-associated T2DM - Related DALYs Burden Among Middle - aged and Elderly Chinese Adults: Results of Age - Period - Cohort Analysis Displaying Trends and Deviations in Rates across Age, Period, and Cohort Dimensions. (A) Age Deviations; (B) Longitudinal Age Curve; (C) Cross - Sectional Age Curve; (D) Period Deviations; (E) Fitted Temporal Trends; (F) Period RR; (G) Cohort Deviations; (H) Cohort RR.

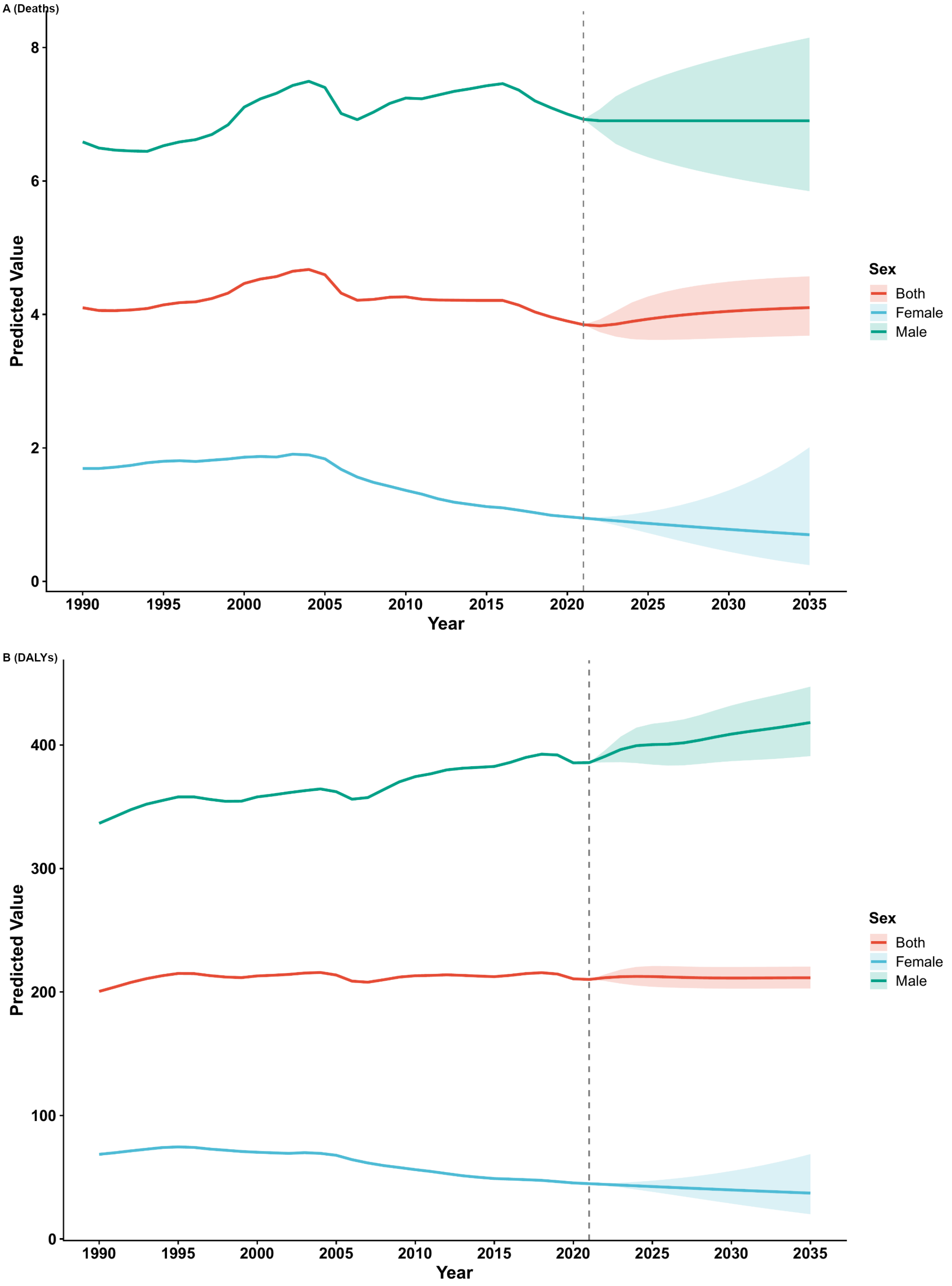

Figure S4 Predicted trends of tobacco-induced low back pain over the next 15 years (2022-2036) (A. Rate of Deaths; B. Rate of DALYs). Lines represent the true and predicted trend; shaded regions represent the predicted trend's 95% CI.

© 2025 He Y. et al.
